# Kt/V reach rate is associated with clinical outcome in incident peritoneal dialysis patients

**DOI:** 10.1080/0886022X.2022.2048854

**Published:** 2022-03-13

**Authors:** Shuang Liu, Lijie Zhang, Shuang Ma, Jing Xiao, Dong Liu, Rui Ding, Zhengyan Li, Zhanzheng Zhao

**Affiliations:** Department of Nephrology, The First Affiliated Hospital of Zhengzhou University, Zhengzhou, China

**Keywords:** Kt/V, reach rate, peritoneal dialysis, clinical outcome

## Abstract

**Background:**

The urea clearance index (Kt/V) is an important index for predicting the clinical outcome of peritoneal dialysis (PD) patients, but it changes with time depending on the clinical condition. This study aimed to investigate the association between the Kt/V reach rate (defined as the percentage of Kt/V measurements that reached ≥ 1.70) and clinical outcome in incident PD patients.

**Methods:**

In this retrospective cohort study, 210 patients were enrolled from the First Affiliated Hospital of Zhengzhou University from 1 January 2013 to 31 October 2019. The target Kt/V reach rate in the first year was applied as the predictor variable. Kaplan-Meier survival curves were drawn to evaluate differences in prognosis. The association between Kt/V reach rate and the composite clinical outcome (death or transfer to hemodialysis) was tested by Cox regression analysis.

**Results:**

The dialysis adequacy group (Kt/V reach rate 3/3 times) and the dialysis intermittent adequacy group (1/3 or 2/3 times) had significantly better clinical outcomes than the dialysis inadequacy group (0/3 times). There was no difference in clinical outcome between the lower-rate group (reach rate 1/3 times) and the higher-rate group (2/3 times). Compared with the dialysis inadequacy group, the dialysis intermittent adequacy group and dialysis adequacy group had significantly lower risks of the composite outcome (HR 0.487, 95% CI 0.244–0.971, *p* = 0.041; HR 0.150, 95% CI 0.043–0.520, *p* = 0.003) in the fully adjusted analysis.

**Conclusion:**

Higher Kt/V reach rates are associated with a better prognosis in incident PD patients.

## Introduction

The approximately 272,000 peritoneal dialysis (PD) patients worldwide account for 11% of the global dialysis patients [1]. According to Chinese insurance claims data, the total number of Chinese dialysis patients was estimated to be 48,482 in 2017 [2]. Dialysis adequacy is recognized as a crucial factor affecting prognosis in continuous ambulatory peritoneal dialysis (CAPD) patients. Evaluating the adequacy of PD plays an important role in improving the quality of life and the survival rate and reducing the technical failure rate.

As a key marker of dialysis adequacy and small solute removal, the importance of the urea clearance index (Kt/V) has been widely acknowledged. The International Society for Peritoneal Dialysis (ISPD) in 2006 [3] and the Kidney Disease Outcome Quality Initiative (K/DOQI) in 2006 [4] both recommended that the minimum target value of Kt/V be 1.70, as this may have tremendous benefit for patients on PD.

However, with the deepening of research, the target value of Kt/V and its relationship with prognosis remains controversial [5,6]. ISPD in 2020 [7] stated that there was a lack of high-quality evidence to prove the impact of Kt/V on survival and mortality, and reaching Kt/V > 1.70 may bring no survival advantage. Therefore, the relationship of Kt/V with clinical outcome needs to be further investigated.

In addition, Kt/V varies profoundly by residual renal function (RRF) and the change of dialysis prescriptions. Previous studies [8,9] using baseline or single-point Kt/V to predict patient outcomes cannot reflect the effect of Kt/V changes on prognosis. The Kt/V reach rate—the percentage of Kt/V measurements that reach the set threshold—may be a more representative index to assess the clinical status of PD patients, especially patients whose Kt/V intermittently meets the standard. At present, the effect of Kt/V reach rate on prognosis is unclear.

In this cohort study, we retrospectively investigated the target reach rate of Kt/V in the first year of incident PD to evaluate its long-term effect on clinical outcome. We hope that our findings can guide physicians to choose the most beneficial treatments for their PD patients.

## Materials and methods

### Participants

This was a single-center retrospective study. All patients were initiated on CAPD for the first time from the PD center of the First Affiliated Hospital of Zhengzhou University from 1 January 2013 to 31 October 2019. Exclusion criteria: (1) age <18 years; (2) duration of PD <1 year; (3) patients on automated PD or intermittent PD; (4) history of kidney transplantation; (5) active systemic inflammatory disease; (6) combined with life-threatening conditions, such as disseminated malignancy or severe organ disease (heart, lung, and liver); (7) loss to follow-up; and (8) no data about the adequacy of dialysis (Kt/V) at 3, 6, or 12 months in the first year of CAPD.

All patients underwent PD catheterization (Tenckhoff tube) in our hospital, using Baxter lactate dialysate (Guangzhou, China) for CAPD, and were prescribed 2-L bags of dialysate containing 1.5 or 2.5% dextrose, with 2–5 exchanges per day. The dialysis prescription was changed according to the individual requirements as determined during the follow-up. All patients were followed until death, transfer to hemodialysis, or the end of the study on 31 October 2020. The time was recorded from the initiation of PD to the endpoint. This study protocol complied with the Declaration of Helsinki and was approved by the ethics committee of the First Affiliated Hospital of Zhengzhou University (approval: 2019-KY-361). Informed consent was not necessary because the data analyzed were all from previous clinical cases, as a retrospective study.

### Clinical data collection

Demographic and clinical information such as sex, age, the underlying cause of end-stage renal disease (ESRD), comorbidities (e.g., diabetes mellitus, hypertension, cardiovascular disease), and the Charlson comorbidity index (CCI) were collected at the initiation of PD. Laboratory data were collected at 3, 6, and 12 months after the start of PD therapy, including body mass index (BMI), systolic blood pressure (SBP), diastolic blood pressure (DBP), hemoglobin, serum albumin, potassium, phosphorus, calcium, intact parathyroid hormone (iPTH), creatinine, uric acid, fasting glucose, total cholesterol, triglyceride, low-density lipoprotein cholesterol (LDL-C), high-density lipoprotein cholesterol (HDL-C), 24-h urine volume, RRF, and normalized protein catabolic rate (nPCR). The transport characteristics of the peritoneal membrane were evaluated by a standard peritoneal equilibration test (PET) [10] and were recorded within 3 months after PD initiation. Cardiovascular disease was defined as a history of angina, class III-IV congestive heart failure, prior myocardial infarction, coronary heart disease, cardiac arrhythmia, cerebrovascular disease, or peripheral vascular disease. RRF was calculated as the mean urea and creatinine clearance from a 24-h urine collection [11]. nPCR was calculated by the formula: nPCR (g/kg/day) = PCR × 0.58/V, PCR (g/day) = 10.76 × (UNA + 1.46), where UNA (g/d) = [24-h urine volume (L) × urine urea nitrogen(mg/dL) + 24-h dialysate volume (L) × dialysate urea nitrogen (mg/dL)]/100. Total body water (V) was determined by Watson’s formula [12]. The cause of ESRD was obtained from the electronic medical record, including pathological diagnosis or clinical diagnosis. When this information was not available, the cause was considered unknown.

### Dialysis adequacy

Dialysis adequacy, for example, total Kt/V, total creatinine clearance (Ccr), and renal Kt/V, were recorded at least at 3, 6, and 12 months after the start of PD. These indices were calculated using the formula recommended in the K/DOQI guidelines [4]. Adequate dialysis was defined as total Kt/V ≥ 1.70, based on the ISPD and K/DOQI guidelines [3,4]. We defined the Kt/V reach rate as the percentage of Kt/V measurements that reached ≥ 1.70 in the first year of PD. All patients were divided into three groups by reach rate: the dialysis inadequacy group (Kt/V reach rate 0/3 times), the dialysis adequacy group (3/3 times), and the dialysis intermittent adequacy group (1/3 or 2/3 times). We divided the dialysis intermittent adequacy group into two subgroups. Patients with a 2/3 Kt/V reach rate were classified as the higher-rate group, whereas those with a 1/3 Kt/V reach rate were classified as the lower-rate group.

### Clinical outcome

The clinical outcome was a composite endpoint including all-cause mortality and conversion to permanent hemodialysis. Clinical outcomes were obtained from the medical records of the PD center or were followed up by telephone at the end of the study. Overall survival time was defined as the months elapsed between the first day of PD and death for any reason, transfer to hemodialysis, or the end of the study in October 2020.

### Statistical analyses

Data were analyzed using SPSS statistics software (version 25.0) and GraphPad Prism 8.0. Time-averaged values of the laboratory indicators were used for statistical analysis. The time-averaged values were the average values of the indicators within the 12-month follow-up. The normality of continuous measurement data was tested by the Kolmogorov-Smirnov test. Continuous variables are expressed as mean ± standard deviation for variables with a normal distribution and median (interquartile range) for variables with a skewed distribution. Categorical variables are expressed as number (*n*) and percentage (%). Multiple-group comparisons were analyzed by analysis of variance or the Kruskal-Wallis nonparametric test. Kaplan-Meier survival curves and log-rank tests were used to evaluate the significance of differences in clinical outcome between groups and subgroups. The Cox proportional hazard regression model was used to determine the associations between different Kt/V categories and clinical outcome. The results are reported as hazard ratios (HRs) with 95% confidence intervals (95% CIs), which are represented graphically in forest plots. A *p* value of < 0.05 was considered statistically significant.

## Results

### Patient characteristics

**Figure 1.**
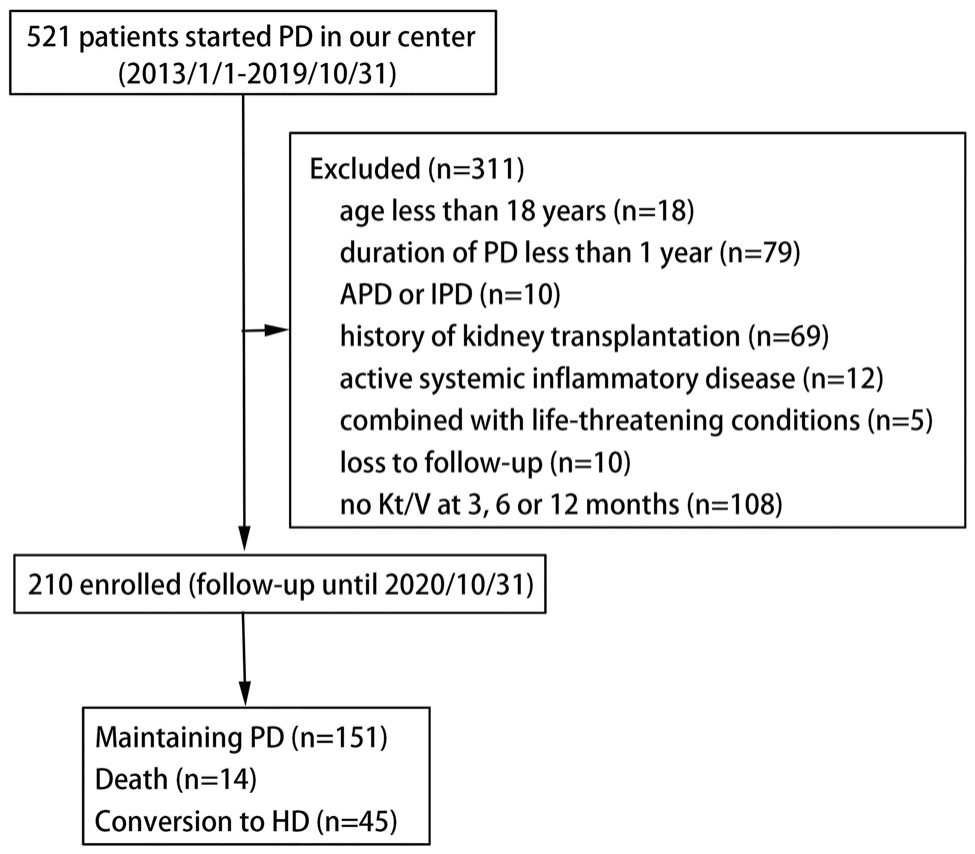
Flow chart of patient selection. APD: automated peritoneal dialysis; HD: hemodialysis; IPD: intermittent peritoneal dialysis; PD: peritoneal dialysis.

From 1 January 2013 to 31 October 2019, a total of 521 patients received CAPD at our hospital. According to the exclusion criteria, 210 patients were enrolled in the study (Figure 1). The mean age of incident patients was 45 (37,51) years at the onset of PD. Among the patients, 122 (58.1%) were males. Their BMI was 22.6 (20.8,24.6) kg/m^2^. The primary causes of ESRD and their proportions were as follows: chronic glomerulonephritis (55.7%), hypertensive renal damage (28.6%), diabetic nephropathy (2.4%), polycystic kidney (8.1%), and unknown (5.2%) (Table 1). Most PD patients had hypertension at the beginning of dialysis (88.6%), some had cardiovascular disease (28.6%), and only 3.3% of all patients had diabetes mellitus. Demographic and clinical characteristics, including the CCI, are shown in Table 1.

**Table 1.** Baseline demographic characteristics for all incident PD patients.

Characteristic	Value
Number of patients (*n*)	210
Age at time of PD (years)	45 (37–51)
Male (*n* %)	122 (58.1)
BMI (kg/m^2^)	22.6 (20.8–24.6)
Cause of ESRD (*n* %)	
Chronic glomerulonephritis	117 (55.7)
Hypertensive renal damage	60 (28.6)
Diabetic nephropathy	5 (2.4)
Polycystic kidney	17 (8.1)
Others	11 (5.2)
Comorbidity (*n* %)	
Hypertension	186 (88.6)
Diabetes mellitus	7 (3.3)
Cardiovascular disease	60 (28.6)
CCI (*n* %)	
0–2	116 (55.2)
3–4	83 (39.5)
≥5	11 (5.2)

Data were express as median (interquartile range) or number (percentage).

BMI: body mass index; CCI: Charlson comorbidity index; ESRD: end-stage renal disease; PD: peritoneal dialysis.

### Comparison of clinical indicators and dialysis adequacy between the three groups

A comparison of clinical indicators and dialysis adequacy between the three groups is presented in Table 2. Most indicators were time-averaged values. There were significant differences in BMI, phosphorus, creatinine, nPCR, RRF, total Kt/V, renal Kt/V, and 24-h urine volume between the three groups. In the dialysis inadequacy group, patients were younger and had higher BMI, higher SBP, lower hemoglobin, higher phosphorus, higher creatinine, lower nPCR, lower RRF, lower total Kt/V, lower renal Kt/V, lower total Ccr, and lower 24-h urine volume than the dialysis adequacy group and dialysis intermittent adequacy group.

**Table 2.** Comparison of clinical parameters and dialysis adequacy among three groups.

Characteristics	Dialysis inadequacy group (*n* = 37)	Dialysis intermittent adequacy group (*n* = 124)	Dialysis adequacy group (*n* = 49)	*p* Value
Age (year)	40.30 ± 11.29	45.50 ± 11.14^a^	45.33 ± 11.91^a^	0.045
BMI (kg/m^2^)*	25.13 (22.79–27.27)	23.00 (20.89–24.35)^a^	21.80 (20.18–23.24)^a,b^	<0.001
SBP (mmHg)*	151.33 (140.67–159.83)	141.67 (135.67–154.00)^a^	142.67 (137.17–148.17)^a^	0.006
DBP (mmHg)*	89.67 (84.67–98.00)	87.33 (82.17–94.50)	89.33 (84.33–95.50)	0.134
Hemoglobin (g/L)*	96.29 ± 14.56	103.44 ± 14.24^a^	108.06 ± 14.26^a^	0.001
Albumin (g/L)*	33.21 ± 4.11	33.83 ± 3.77	34.34 ± 3.74	0.402
Potassium (mmol/L)*	4.33 (3.86–4.62)	4.23 (3.93–4.59)	4.08 (3.79–4.48)	0.285
Phosphorus (mmol/L)*	1.85 (1.68–2.04)	1.65 (1.45–1.97)^a^	1.54 (1.33–1.73)^a,b^	<0.001
Calcium (mmol/L)*	2.15 (2.04–2.25)	2.14 (2.03–2.24)	2.21 (2.11–2.33)	0.082
iPTH (pg/mL)*	210.77 (123.06–338.32)	225.95 (140.44–333.80)	227.73 (114.49–400.00)	0.798
Creatinine (μmol/L)*	1080.67 (1002.50–1245.42)	937.08 (770.33–1048.50)^a^	760.67 (626.35–854.48)^a,b^	<0.001
Uric acid (μmol/L)*	364.00 (339.33–408.67)	377.83 (327.42–412.25)	337.67 (299.00–379.67)^a,b^	0.004
Fasting glucose (mmol/L)*	4.20 (3.88–4.61)	4.36 (3.95–4.85)	4.15 (4.01–4.71)	0.348
Total cholesterol(mmol/L)*	4.09 (3.61–4.34)	4.18 (3.64–4.90)	4.43 (3.80–4.96)	0.168
Triglyceride (mmol/L)*	1.02 (0.70–1.42)	1.12 (0.90–1.39)	1.13 (0.91–1.36)	0.192
LDL-C (mmol/L)*	2.43 (2.04–2.82)	2.69 (2.20–3.26)	2.78 (2.19–3.19)	0.220
HDL-C (mmol/L)*	1.14 (0.88–1.44)	1.13 (1.00–1.35)	1.16 (0.94–1.50)	0.802
nPCR (g/kg/d)*	1.00 (0.86–1.13)	1.18 (1.00–1.40)^a^	1.32 (1.07–1.62)^a.b^	<0.001
RRF (mL/min/1.73 m^2^)*	1.84 (1.10–2.64)	2.71 (1.55–3.89)^a^	3.97 (2.93–5.90)^a,b^	<0.001
D/Pcr	0.72 (0.68–0.85)	0.73 (0.65–0.81)	0.72 (0.63–0.86)	0.740
Total Kt/V (per week)*	1.38 (1.27–1.48)	1.71 (1.62–1.87)^a^	2.15 (1.98–2.37)^a,b^	<0.001
Renal Kt/V (per week)*	0.34 ± 0.19	0.62 ± 0.30^a^	1.03 ± 0.44^a,b^	<0.001
Total Ccr [L/(w*1.73 m^2^)]*	60.67 (51.35–66.68)	61.05 (52.96–73.35)	75.68 (64.73–93.63)^a,b^	<0.001
Urine volume (L/24 h)*	0.88 (0.58–1.13)	1.10 (0.70–1.63)^a^	1.75 (1.22–2.17)^a,b^	<0.001

Data were express as mean ± SD or median (interquartile range).

Dialysis inadequacy group: patients with a Kt/V reach rate of 0/3 times; Dialysis intermittent adequacy group: patients with a Kt/V reach rate of 1/3 or 2/3 times; Dialysis adequacy group: patients with a Kt/V reach rate of 3/3 times. Kt/V reach rate: the percentage of Kt/V measurements that reached ≥ 1.70 in the first of PD.

BMI: body mass index; Ccr: creatinine clearance; D/Pcr: dialysate/plasma creatinine ratio at 4 h; DBP: diastolic blood pressure; HDL-C: high-density lipoprotein cholesterol; iPTH: intact parathyroid hormone; Kt/V: urea clearance index; LDL-C: low-density lipoprotein cholesterol; nPCR: normalized protein catabolic rate; RRF: residual renal function; SBP: systolic blood pressure.

Comparison of dialysis inadequacy group, ^a^*p* < 0.05; comparison of dialysis intermittent adequacy group, ^b^*p* < 0.05.

*Time-averaged values.

### Kaplan-Meier survival analysis of groups and subgroups

**Figure 2.**
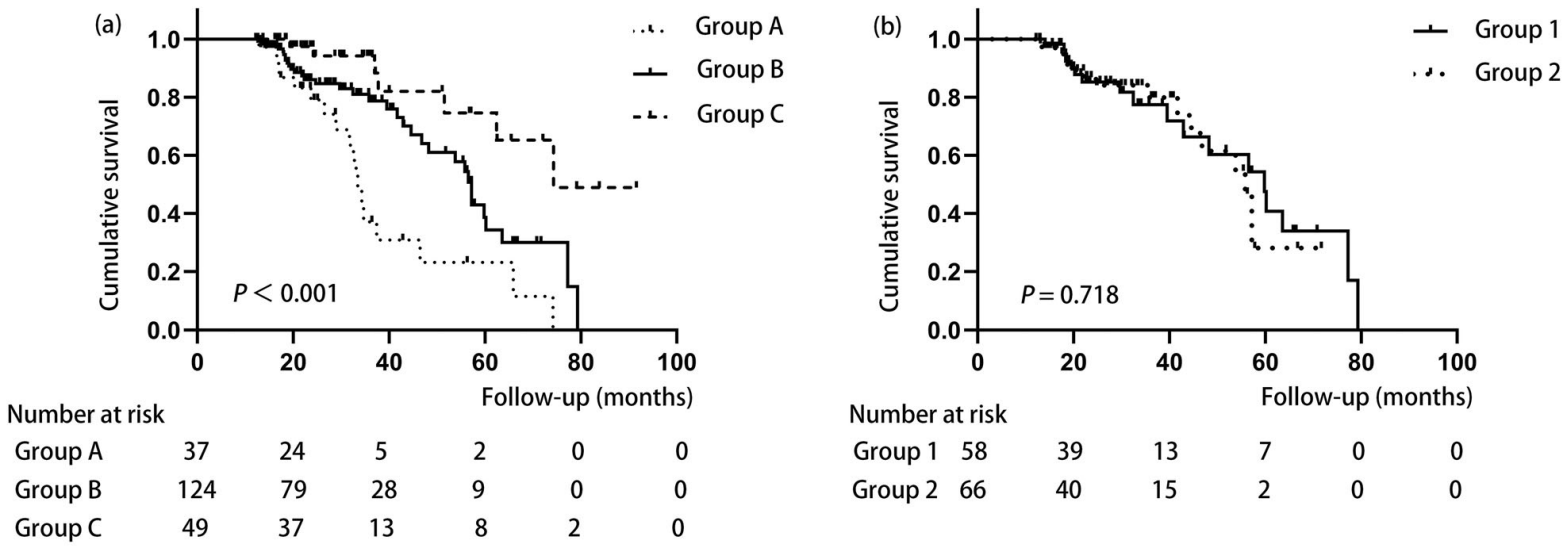
(a) Kaplan-Meier curves of the three groups for overall survival. Group A (Dialysis inadequacy group): patients with a Kt/V reach rate of 0/3 times; Group B (Dialysis intermittent adequacy group): patients with a Kt/V reach rate of 1/3 or 2/3 times; Group C (Dialysis adequacy group): patients with a Kt/V reach rate of 3/3 times. (b) Kaplan-Meier curve of the two intermittent subgroups for overall survival. Group 1 (lower-rate group): patients with a 1/3 Kt/V reach rate; Group 2 (higher-rate group): patients with a 2/3 Kt/V reach rate. Kt/V reach rate: the percentage of Kt/V measurements that reached ≥ 1.70 in the first year of PD.

The overall median follow-up time was 24.75 months (range: 12.20–91.57 months). During follow-up, 59 (28.10%) patients reached the composite endpoint, including 14 (6.67%) who died and 45 (21.43%) who were transferred to hemodialysis. The causes of death were cardiovascular disease (3 patients), cerebrovascular accidents (2), systemic failure (2), infection (1), hyperkalemia (1), and unknown (5). The causes of transfer to hemodialysis were inadequate dialysis in 27 patients, PD-related infections in 15 patients, and technical failure in 3 patients. For the composite endpoint event, Kaplan-Meier survival curves revealed that the overall survival was significantly better in the dialysis adequacy group and dialysis intermittent adequacy group than in the dialysis inadequacy group. Further subgroup analysis showed no significant difference in overall survival between the lower-rate group and the higher-rate group (Figure 2).

### Cox regression analysis prognostic factors

The results of Cox hazard regression analysis for the clinical outcome (mortality or technical survival) are summarized in Table 3 and are displayed in a forest plot. Univariate Cox regression analysis indicated that sex, time-averaged albumin, time-averaged phosphorus, time-averaged HDL-C, and Kt/V reach rate were associated with the clinical outcome. By multivariate Cox regression analysis, female sex (HR = 0.397, 95% CI = 0.184–0.856, *p* = 0.018), time-averaged albumin (HR = 0.925, 95% CI = 0.859–0.996, *p* = 0.040), time-averaged HDL-C (HR = 0.273, 95% CI = 0.078–0.961, *p* = 0.043), and Kt/V reach rate were independent protective factors. Patients with a 3/3 Kt/V reach rate had the highest overall survival. Compared with the dialysis inadequacy group, the dialysis adequacy group had an 85% lower risk of the composite endpoint (HR = 0.150, 95% CI = 0.043–0.520, *p* = 0.003) and the dialysis intermittent adequacy group, 51.3% (HR = 0.487, 95% CI = 0.244–0.971, *p* = 0.041).

**Table 3.** Cox hazard regression analysis of characteristics and clinical outcome.

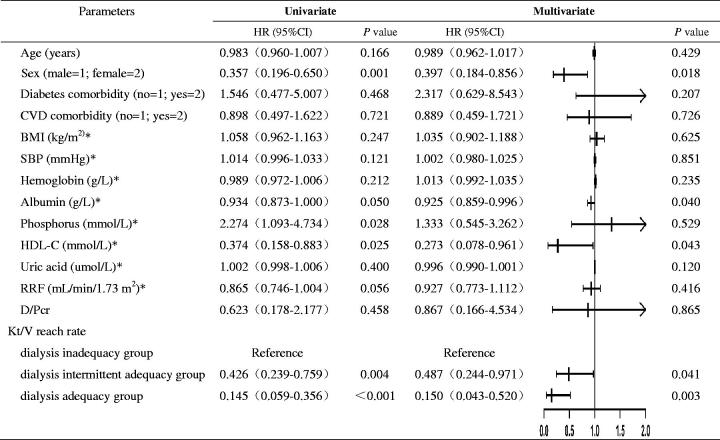					

Data were expressed as HR and 95% confidence interval (95% CI).

Dialysis inadequacy group: patients with a Kt/V reach rate of 0/3 times; Dialysis intermittent adequacy group: patients with a Kt/V reach rate of 1/3 or 2/3 times; Dialysis adequacy group: patients with a Kt/V reach rate of 3/3 times. Kt/V reach rate: the percentage of Kt/V measurements that reached ≥ 1.70 in the first of PD.

BMI: body mass index; CVD: cardiovascular disease; D/Pcr: dialysate/plasma creatinine ratio at 4 h; HDL-C: high-density lipoprotein cholesterol; Kt/V: urea clearance index; RRF: residual renal function; SBP: systolic blood pressure.

*Time-averaged values.

## Discussion

In this study, we examined the association of the total Kt/V reach rate with clinical outcomes (death and technical survival) among incident PD individuals. Patients in the dialysis adequacy group and the dialysis intermittent adequacy group had a significantly better prognosis than those in the dialysis inadequacy group. We confirmed the impact of Kt/V and the target Kt/V reach rate on clinical outcome. This is the first study to use the target Kt/V reach rate to predict the prognosis of PD patients.

The relationship between Kt/V and prognosis remains controversial [5], including studies from China. Szeto et al. [13] found that Kt/V was an independent predictor of clinical outcome only for new CAPD cases, not for prevalent cases. Lo et al. [14] showed that Kt/V had no effect on clinical outcome, but clinical problems and severe anemia were significantly increased in patients with total Kt/V below 1.70. While, another large multicenter study from China [15] confirmed that Kt/V was independently associated with one-year mortality in a prognostic model. Similar result was observed in a recent study from Taiwan [9] that lower Kt/V was a risk factor for mortality among PD patients. Differences in the study population, study design, methods, length of follow-up, outcome definitions, and level of baseline RRF can partially account for the inconsistent results. In this study, we used Kt/V reach rate as the grouping variable to analyze the relationship between Kt/V and prognosis. Our study is consistent with the finding by Szeto et al. [13] that patients in the dialysis adequacy group had better clinical outcomes than those in the dialysis inadequacy group. Furthermore, the Kt/V reach rate was associated with prognosis.

There are several possible reasons for the relationship between Kt/V and prognosis. First, Kt/V is an important quantitative index to evaluate small solute clearance, and is known as one of the major determinants of dialysis adequacy. Second, Kt/V has a protective effect on RRF preservation [16]. Patients with better RRF have a better prognosis [17]. Third, it has been reported that high Kt/V can reduce the incidence of peritonitis [8]. Peritonitis is a major cause of death and technique failure in PD patients. Fourth, Kt/V is inversely correlated with over-hydration [18], promoting the removal of fluid and sodium. Last but not least, there is a positive association between Kt/V and hemoglobin [16], which can improve the prognosis. Unfortunately, we did not see a difference in clinical outcome between the lower-rate group and the higher-rate group. A plausible explanation is that good clinical status weakens the effect of Kt/V on prognosis, since there was no difference in clinical indicators between the two groups except BMI, creatinine, and RRF (data not shown).

Consistent with previous research results, we found a relationship between higher albumin or HDL-C and better prognosis. Albumin is an indicator of nutritional status. Hypoalbuminemia is associated with decreased immune function, an increased risk of peritonitis, volume overload, and RRF loss [19]. Previous studies have shown that hypoalbuminemia is a valid predictor of mortality and technique failure [9,15]. HDL-C can promote reverse cholesterol transport, protect endothelial cells, and have anti-inflammatory and antioxidant effects on the development of atherosclerosis [20]. Yao et al. [21] suggest that HDL-C is correlated with a reduction in adverse cardiac and cerebral events in CAPD, possibly leading to a better prognosis. Diabetes can affect the prognosis of PD patients [8,15], but our study did not find a significant correlation. One possible reason is that comorbidities only represented the baseline situation, and the proportion of patients with diabetes was low (3.3%), leading to negative results. We also did not find a relationship between age and prognosis. It may be that the age of PD patients in this study was relatively narrowly distributed and relatively young. This probably represented a type II error.

This study also revealed that female sex was an independent protective factor. The risk in female patients was approximately 60% lower than that in male patients, in line with Kitterer et al. [22]. This could be attributed to more careful and standardized operation procedures, better compliance, and less peritonitis in females than males [22]. In addition, as the body surface of females is genetically smaller than that of males, a lower dialysis dose was needed to achieve sufficient dialysis, which was beneficial for prognosis [23].

Patients in the dialysis inadequacy group were younger, had higher BMI, had poorer RRF, and had significantly more clinical problems, such as higher SBP, lower hemoglobin, higher blood phosphorus, and lower nPCR. Kt/V is negatively correlated with BMI and positively correlated with RRF and nPCR [16]. Lower hemoglobin and higher blood phosphorus are presumably related to renal endocrine dysfunction and a deterioration of calcium-phosphate metabolism regulation, which are results of poor RRF. RRF is important in PD patients [17]. However, time-averaged RRF was not an independent prognostic factor in our study. This may be related to the decline in RRF with dialysis time, so the protective effect of RRF gradually disappeared. Wang et al. [24] found that RRF decline in the first year of PD was an independent predictor of prognosis during the first 3 years of PD therapy, while after more than 3 years, RRF decline no longer correlated with clinical outcomes. Another notable point is that patients were younger in the dialysis inadequacy group. Some plausible explanations are as follows: First, young people have higher resting energy expenditure. Resting energy expenditure is negatively correlated with total Kt/V [25]. Second, younger patients had poor RRF at the start of PD due to the late initiation of dialysis. This delay in the initiation of dialysis may have been due to poor psychological acceptance of dialysis and a reluctance to initiate dialysis, or to their strong tolerance to toxins. Third, young people have worse compliance. An irregular lifestyle and poor dietary control can affect the therapeutic effect of PD. Some young patients refuse to increase the dialysis dose to avoid impacting their social lives or work during the dialysis interphase.

This study confirmed the long-term impact of dialysis adequacy and Kt/V reach rate in the first year on the prognosis of PD patients. Monitoring the change of Kt/V had significant value during the period of PD therapy. In our study, it had a longer observational period than the 2–3 years of most short-term observational studies. At least 27.62% (58/210) of patients were followed up for more than 3 years, and the maximum follow-up duration was 91 months. Our study does have several limitations. First, it was a single-center study with relatively few patients. Second, it was a retrospective study, so there may be confounding factors. Third, we excluded patients who had missing Kt/V data at 3, 6, or 12 months in the first year of CAPD and patients with endpoint events within 1 year to exclude the ‘sicker’ patients, which may have caused selection bias. Moreover, the proportion of patients with diabetes was low (3.3%), so the results may not be applicable to PD patients with diabetes. Finally, most of the 210 patients were in the dialysis intermittent adequacy group, leading to a bias from the uneven distribution of patients between the different groups. Multicenter, large-sample prospective cohort studies are still needed to confirm the effect of total Kt/V on prognosis and the role of RRF.

## Conclusion

The target Kt/V reach rate has a long-term effect on the clinical outcome of PD patients. A sustained Kt/V ≥ 1.70 is crucial for maintaining a better prognosis in prevalent PD patients. Our findings can provide a reference for the prognosis and adjustment of dialysis prescriptions of PD patients.

## Data Availability

All data generated or analyzed during this study are included in this article. Further enquiries can be directed to the corresponding author.

## References

[1] Li PK, Chow KM, Van de Luijtgaarden MW, et al. Changes in the worldwide epidemiology of peritoneal dialysis. Nat Rev Nephrol. 2017;13(2):90–103. 28029154 10.1038/nrneph.2016.181

[2] Yang C, Yang Z, Wang J, et al. Estimation of prevalence of kidney disease treated with dialysis in China: a study of insurance claims data. Am J Kidney Dis. 2021;77(6):889–897.e1. 33421457 10.1053/j.ajkd.2020.11.021

[3] Lo WK, Bargman JM, Burkart J, et al. Guideline on targets for solute and fluid removal in adult patients on chronic peritoneal dialysis. Perit Dial Int. 2006;26(5):520–522. 16973505

[4] Gilmore J. KDOQI clinical practice guidelines and clinical practice recommendations–2006 updates. Nephrol Nurs J. 2006;33(5):487–488. 17044433

[5] Bargman JM. We use Kt/V urea as a measure of adequacy of peritoneal dialysis. Semin Dial. 2016;29(4):258–259. 27081745 10.1111/sdi.12504

[6] Rees L. Assessment of dialysis adequacy: beyond urea kinetic measurements. Pediatr Nephrol. 2019;34(1):61–69. 29582148 10.1007/s00467-018-3914-6 PMC6244854

[7] Brown EA, Blake PG, Boudville N, et al. International society for peritoneal dialysis practice recommendations: prescribing high-quality goal-directed peritoneal dialysis. Perit Dial Int. 2020;40(3):244–253. 32063219 10.1177/0896860819895364

[8] Pulliam J, Li NC, Maddux F, et al. First-year outcomes of incident peritoneal dialysis patients in the United States. Am J Kidney Dis. 2014;64(5):761–769. 24927898 10.1053/j.ajkd.2014.04.025

[9] Chen HL, Tarng DC, Huang LH. Risk factors associated with outcomes of peritoneal dialysis in Taiwan: an analysis using a competing risk model. Medicine. 2019;98(6):e14385. 30732176 10.1097/MD.0000000000014385 PMC6380716

[10] Twardowski ZJ. Clinical value of standardized equilibration tests in CAPD patients. Blood Purif. 1989;7(2–3):95–108. 2663040 10.1159/000169582

[11] van Olden RW, Krediet RT, Struijk DG, et al. Measurement of residual renal function in patients treated with continuous ambulatory peritoneal dialysis. JASN. 1996;7(5):745–750. 8738810 10.1681/ASN.V75745

[12] Watson PE, Watson ID, Batt RD. Total body water volumes for adult males and females estimated from simple anthropometric measurements. Am J Clin Nutr. 1980;33(1):27–39. 6986753 10.1093/ajcn/33.1.27

[13] Szeto CC, Wong TY, Leung CB, et al. Importance of dialysis adequacy in mortality and morbidity of Chinese CAPD patients. Kidney Int. 2000;58(1):400–407. 10886588 10.1046/j.1523-1755.2000.00179.x

[14] Lo WK, Ho YW, Li CS, et al. Effect of Kt/V on survival and clinical outcome in CAPD patients in a randomized prospective study. Kidney Int. 2003;64(2):649–656. 12846762 10.1046/j.1523-1755.2003.00098.x

[15] Cao XY, Zhou JH, Cai GY, et al. Predicting one-year mortality in peritoneal dialysis patients: an analysis of the China peritoneal dialysis Registry. Int J Med Sci. 2015;12(4):354–361. 26019685 10.7150/ijms.11694 PMC4445016

[16] Qin A, Liu X, Yin X, et al. Normalized protein catabolic rate is a superior nutritional marker associated with dialysis adequacy in continuous ambulatory peritoneal dialysis patients. Front Med. 2020;7:603725. 10.3389/fmed.2020.603725 PMC7835658 33511142

[17] Wang AY, Lai KN. The importance of residual renal function in dialysis patients. Kidney Int. 2006;69(10):1726–1732. 16612329 10.1038/sj.ki.5000382

[18] Kwan BC, Szeto CC, Chow KM, et al. Bioimpedance spectroscopy for the detection of fluid overload in Chinese peritoneal dialysis patients. Perit Dial Int. 2014;34(4):409–416. 24385329 10.3747/pdi.2013.00066 PMC4079487

[19] Kiebalo T, Holotka J, Habura I, et al. Nutritional status in peritoneal dialysis: nutritional guidelines, adequacy and the management of malnutrition. Nutrients. 2020;12(6):1715. 10.3390/nu12061715 PMC7352713 32521626

[20] Assmann G, Gotto AM Jr. HDL cholesterol and protective factors in atherosclerosis. Circulation. 2004;109(23 Suppl 1):III8–14. 15198960 10.1161/01.CIR.0000131512.50667.46

[21] Yao C, Zhou L, Huang Q. The occurrence and potential predictive factors of major adverse cardiac and cerebral events in end-stage renal disease patients on continuous ambulatory peritoneal dialysis: a prospective cohort study. Medicine . 2021;100(10):e24616. 33725825 10.1097/MD.0000000000024616 PMC7969313

[22] Kitterer D, Segerer S, Braun N, et al. Gender-specific differences in peritoneal dialysis. Kidney Blood Press Res. 2017;42(2):276–283. 28531889 10.1159/000477449

[23] Radunz V, Pecoits-Filho R, Figueiredo AE, et al. Impact of glucose exposure on outcomes of a nation-wide peritoneal dialysis cohort – results of the BRAZPD II cohort. Front Physiol. 2019;10(150):150. 30890947 10.3389/fphys.2019.00150 PMC6411763

[24] Wang J, Xie X, Yan X, et al. A fast decline of residual renal function in the first year is a predictor for early withdrawal from peritoneal dialysis in non-diabetic patients. Kidney Blood Press Res. 2019;44(1):12–21. 30808853 10.1159/000497807

[25] Xu T, Xie J, Wang W, et al. Resting energy expenditure: a valuable predictor for KT/Vurea in peritoneal dialysis patients. Clin Nephrol. 2017;88(9):124–131. 28766494 10.5414/CN109042

